# Embodied Space in Early Blind Individuals

**DOI:** 10.3389/fpsyg.2012.00272

**Published:** 2012-08-01

**Authors:** Virginie Crollen, Olivier Collignon

**Affiliations:** ^1^Centre de Neuroscience Système et Cognition, Institut de Recherche en Sciences Psychologiques, Université Catholique de LouvainLouvain, Belgium; ^2^Centre de Recherche en Neuropsychologie et Cognition, Université de MontréalMontréal, QC, Canada; ^3^Centre for Mind/Brain Science, University of TrentoTrento, Italy

The study of visually deprived individuals offers a unique opportunity to investigate the role that vision plays in shaping how we process our surrounding space. The visual system typically provides the most accurate and reliable spatial information about our surroundings and therefore is usually considered as the primary sense when spatial processing is at play. One of the best examples of such visual dominance in space perception comes from experiments showing that when a sound is accompanied by a visual stimulus at a different location, people tend to perceive this sound incorrectly at the same position as the visual stimulus (Pick et al., 1969). This “ventriloquist” effect occurs because the brain affords more weight to visual information in localizing the audiovisual event, thus inducing a “visual capture” of acoustic space (Alais and Burr, 2004).

It was first thought that visual deprivation might be detrimental to the development of spatial abilities in the remaining modalities since vision may be required to calibrate the other sensory systems (Axelrod, 1959; Rock and Halper, 1969; Warren and Cleaves, 1971). Interestingly, this does not appear to be the case since several studies have shown that blind people are usually as good as and often better than normal sighted controls (SCs) in the processing of non-visual spatial inputs (Lessard et al., 1998; see Collignon et al., 2009a for review). The recurrent hypothesis to explain such findings is that vision loss is partly offset by an increased use of the remaining senses (Wong et al., 2011) which triggers enhancement in their efficiency concomitantly to compensatory brain reorganization processes (Gougoux et al., 2005; Collignon et al., 2011). If this may be a part of the story, another possibility that we want to address in the present paper is that aside quantitative differences between sighted and blind people in their perceptual skills, visual deprivation may result in qualitatively different ways of processing non-visual information (Eimer, 2004). While sighted people may automatically process spatial information in an external spatial frame of reference.^1^ Early blind (EB) participants may preferentially use an internal coordinate system.^2^

Bradshaw et al. (1986) were the first to suggest a qualitative difference in the way EB individuals process touch. In this study, a rod was placed within a shorter pipe. EB and SC participants were asked to slide the rod within the pipe until the rod extremities were judged equidistant from the ends of the pipe. Results demonstrated that SC placed the midline of the pipe slightly to the left of the true midpoint (leftward bias or pseudo-neglect; see Jewell and McCourt, 2000 for a review) with hands placed in parallel or crossed over the body midline. EB participants, in contrast, showed a leftward bias with hands in parallel (see also Sampaio et al., 1995) but a rightward bias with the arms crossed. Even if this effect was only elusively discussed, authors nonetheless interpreted it as reflecting a more internal representation of space in EB. According to the view that the right hemisphere plays a dominant role in attentional control, the leftward bias shown by sighted individuals may be due to the fact that the right hemisphere bias attention to the left visual space so that rods appear longer in the control lateral left hemifield. The reversed pseudo-neglect effect presented by the EB in the crossed posture may in contrast indicate that the right hemisphere of these participants actually affords more attention to the contralateral tactile space therefore leading to an overestimation of the side of space where the left hand is placed.

More recently, Röder et al. (2004) brought new and more compelling evidence in support of this idea. In their temporal order judgment (TOJ) task, participants were asked which of the two hands received a tactile stimulus first. As expected, SC were less accurate with crossed than with uncrossed hands (Yamamoto and Kitazawa, 2001; Shore et al., 2002). This is accounted by the fact that tactile stimuli are not only represented in an anatomical reference frame but are automatically remapped into external spatial coordinates, inducing a conflict between somatotopic and external spatial codes when the hands are crossed over the body midline (Pavani et al., 2000; Kitazawa, 2002; Shore et al., 2002; Azañón and Soto-Faraco, 2008; Azañón et al., 2010a). By contrast, crossing the hands did not lead to a general decrement in EB tactile discrimination performance (Röder et al., 2004) suggesting that the automatic external remapping process of touch was not innate but rather depended on early visual experience (see also Bremner et al., 2008). This idea was later supported by an electroencephalographic study. While the detection of deviant tactile stimuli on the hand induced event-related potentials that varied in crossed when compared to uncrossed condition in SC, changing the posture of the hand had no influence on the EB brain activity (Röder et al., 2008).

The lower incidence of using an external reference frame in EB individuals has also been observed in tasks investigating the multisensory control of action (Simon effect: Röder et al., 2007), the processing of numbers (SNARC effect: Crollen et al., 2011), and spatial navigation (Vecchi et al., 2004; Noordzij et al., 2006). When required to press a left or right response key depending on the bandwidth of a sound presented from a left or right loudspeaker, EB reacted as late blind (LB) and SC in an uncrossed hand posture: they performed better when the spatial localization of the sound was compatible with the spatial localization of the response key (i.e., Simon effect). In contrast, when participants performed the task with crossed hands EB performed more rapidly than their sighted peers and, interestingly, presented a reversal of the Simon effect while LB and SC still showed a classic Simon effect (Röder et al., 2007). The presentation of a sensory stimulus to SC and LB therefore primes the response key compatible with the location of the stimulus in external space, regardless of which anatomical hand is used to press it. In EB, in contrast, the sensory stimulus primes the anatomical hand congruent with the location of the stimulus, regardless of where in space that hand is placed. SC, LB, and EB also presented a similar behavioral pattern when performing a numerical comparison task in an uncrossed hands posture. They responded faster when a left response was required for numbers smaller than five and when a right response was required for numbers larger than five (i.e., SNARC effect). As in the Simon task, however, crossing the hands resulted in a reversal of the SNARC effect in EB participants only (Crollen et al., 2011). The fact that LB and SC participants were similarly affected by crossing the hands indicates that once an external frame of reference is acquired it will continue to be used even though visual information may no longer be available (Röder et al., 2004, 2007; Crollen et al., 2011). Finally, differences between blind and sighted subjects have also been highlighted in spatial navigation tasks. While tasks requiring the use of an egocentric reference frame (i.e., route-knowledge) are performed equally well by SC and EB, tasks requiring the use of an allocentric reference frame (i.e., survey knowledge) are performed less well by the EB than by the SC (Vecchi et al., 2004; Noordzij et al., 2006).

At this stage, one may wonder why sighted individuals automatically remap touch in external coordinates since it can lead to confusion and slow down their reaction times (RT) when discriminating tactile information. This automatic remapping from somatotopic to external space is actually very effective to provide a common framework to coordinate and integrate spatial information obtained through touch with spatial information obtained through other sensory modalities, such as vision or audition which are coded by default in external spatial coordinates. This is particularly critical since the hands move constantly in the peri-personal space as different postures are adopted. The default use of an anatomically anchored reference system in EB may therefore actually prevent the effective integration of different sensory modalities in a multisensory integration task.

In a recent study, EB, LB, and SC groups were required to lateralize auditory, tactile, and audio-tactile stimuli either with the hands uncrossed or crossed over the body midline (Collignon et al., 2009b). While performance in the tactile condition replicated the pattern of results found in previous studies (greater detrimental effect of the crossed posture in LB and SC relative to EB), the results of the auditory and audio-tactile conditions showed a greater detrimental effect of the crossed posture in EB. As mentioned earlier, when EB lateralize tactile stimuli in crossed posture they do not remap the proprioceptive information onto an external spatial frame of reference and therefore do not present the conflict between body-centered and external coordinates that is present in SC or even in LB (Röder et al., 2004). EB therefore process spatial tactile information faster than their sighted peers. In contrast, the absence of automatic external remapping of touch in EB actually prevents these participants from efficiently matching the external sound location and the anatomical coordinate of the responding (auditory condition) or stimulated (audio-tactile condition) hand. The conflict created by crossing the hands is therefore more disrupting in EB than in SC or LB in the auditory and audio-tactile condition (see also Röder et al., 2007, Experiment 2). In other words, the absence of automatic activation of an external reference frame for perception and action in EB may impair multisensory integration and action control when there is a conflict between anatomical and external reference frames, for instance, when a sound has to be integrated with a touch in a hand-crossed posture (Collignon et al., 2009b).

In sum, developmental vision appears to trigger the development of the automatic recoding of sensory-perception/motor-control in an external space. Our opinion is that some of the advantages/deficits observed in EB (e.g., faster/slower RTs to non-visual events) might be explained, at least in part, by such qualitative changes in the way they process non-visual spatial information. For example, one of the most recurrent finding in the blind literature is the observation of faster RT to non-visual spatial targets in EB when compared to SC (e.g., Kujala et al., 1997; Hötting et al., 2004; Collignon et al., 2006, 2009b; Collignon and De Volder, 2009). Since the automatic external remapping process appears to occur between 100 and 360 ms (Azañón and Soto-Faraco, 2008; Heed and Röder, 2010; Overvliet et al., 2011), blind participants who do not automatically remap tactile/spatial information in external space may not only be more resistant to conflict created by crossing hand posture but may also process spatial information some hundreds of milliseconds faster than sighted individuals. Indeed, in the TOJ (Röder et al., 2004), SIMON (Röder et al., 2007), and SNARC (Crollen et al., 2011) experiments described above, EB participants consistently showed faster RT to non-visual targets in the uncrossed posture. Indirect support of the idea that EB may somehow “skip” the external remapping computational step also comes from our observation that transcranial magnetic stimulation over the right intra-parietal sulcus (where the external remapping seems to occur: Makin et al., 2007; Azañón et al., 2010b) disrupted the spatial processing of sounds only in SC but not in EB (Collignon et al., 2009c).

Interestingly, using either an internal or an external frame of reference appears to facilitate performance on different tasks. While the default use of an internal reference frame leads to better performance in tactile lateralization task in EB, the use of an external frame of reference is more adapted for spatial navigation (Noordzij et al., 2006), multisensory integration, and the control of action toward external auditory sources in peri-personal space (Röder et al., 2007; Collignon et al., 2009b). It is however important to note that the spontaneous tendency to organize the environment through internal coordinates in EB does not mean that they are incapable of constructing an external coordinate system (see Eardley and van Velzen, 2011) but this form of encoding is less automatic than the anatomical one in this population.
